# An Introduction to 3D Bioprinting: Possibilities, Challenges and Future Aspects

**DOI:** 10.3390/ma11112199

**Published:** 2018-11-06

**Authors:** Željka P. Kačarević, Patrick M. Rider, Said Alkildani, Sujith Retnasingh, Ralf Smeets, Ole Jung, Zrinka Ivanišević, Mike Barbeck

**Affiliations:** 1 Department of Anatomy Histology, Embryology, Pathology Anatomy and Pathology Histology, Faculty of Dental Medicine and Health, University of Osijek, 31000 Osijek, Croatia; 2 Botiss Biomaterials, Hauptstraße 28, 15806 Zossen, Germany; patrick.rider@botiss.com (P.M.R.); mike.barbeck@icloud.com (M.B.); 3 Department of Biomedical Engineering, Faculty of Applied Medical Sciences, German-Jordanian University, 11180 Amman, Jordan; saidkildani@gmail.com; 4 Institute for Environmental Toxicology, Martin-Luther-Universität, Halle-Wittenberg and Faculty of Biomedical Engineering, Anhalt University of Applied Science, 06366 Köthen, Germany; sujiroshi@gmail.com; 5 Department of Oral and Maxillofacial Surgery, University Hospital Hamburg-Eppendorf, 20246 Hamburg, Germany; r.smeets@uke.de; 6 Department of Oral Maxillofacial Surgery, Division of Regenerative Orofacial Medicine, University Medical Center Hamburg-Eppendorf, 20246 Hamburg, Germany; ol.jung@uke.de; 7 Department of Dental Medicine, Faculty of Dental Medicine and Health, University of Osijek, 31000 Osijek, Croatia; zrinkaivan@gmail.com; 8 BerlinAnalytix GmbH, 12109 Berlin, Germany

**Keywords:** additive manufacturing, 3D scaffolds, inkjet, extrusion, stereolithography, laser-assisted, rapid prototyping

## Abstract

Bioprinting is an emerging field in regenerative medicine. Producing cell-laden, three-dimensional structures to mimic bodily tissues has an important role not only in tissue engineering, but also in drug delivery and cancer studies. Bioprinting can provide patient-specific spatial geometry, controlled microstructures and the positioning of different cell types for the fabrication of tissue engineering scaffolds. In this brief review, the different fabrication techniques: laser-based, extrusion-based and inkjet-based bioprinting, are defined, elaborated and compared. Advantages and challenges of each technique are addressed as well as the current research status of each technique towards various tissue types. Nozzle-based techniques, like inkjet and extrusion printing, and laser-based techniques, like stereolithography and laser-assisted bioprinting, are all capable of producing successful bioprinted scaffolds. These four techniques were found to have diverse effects on cell viability, resolution and print fidelity. Additionally, the choice of materials and their concentrations were also found to impact the printing characteristics. Each technique has demonstrated individual advantages and disadvantages with more recent research conduct involving multiple techniques to combine the advantages of each technique.

## 1. Introduction

Bioprinting is a subcategory of additive manufacturing (AM), also known as three-dimensional (3D) printing. It is defined as the printing of structures using viable cells, biomaterials and biological molecules [1,2]. Bioprinting must produce scaffolds with a suitable microarchitecture to provide mechanical stability and promote cell ingrowth whilst also considering the impact of manufacture on cell viability; for instance, chemical cytotoxicity caused by the use of solvents or pressure-induced apoptotic effect produced during the extrusion of material. A significant benefit of bioprinting is that it prevents homogeneity issues that accompany post-fabrication cell seeding, as cell placement is included during fabrication.

The advantage of homogeneously distributed cell-laden scaffolds has been demonstrated by faster integration with the host tissue, lower risk of rejection and most importantly, uniform tissue growth in vivo [3,4,5,6]. Conventional cell seeding techniques are either static or dynamic, and while the latter one results in better seeding efficiency and cell penetration into the scaffold, it is known affect cell morphology [7]. 

Immediate vascularization of the implanted scaffolds is highly critical [8,9]. With proper vascularization, the scaffolds are provided with an influx of oxygen/nutrients and an efflux of carbon dioxide/by-products; preventing core necrosis. Vascularization also supports the implants with remodelling [10]. Bioprinting techniques have been employed to fabricate microvascular-like structures and have the potential to position endothelial cells within the 3D structures as a prevascularization step prior to implantation [11].

**Figure 1:**
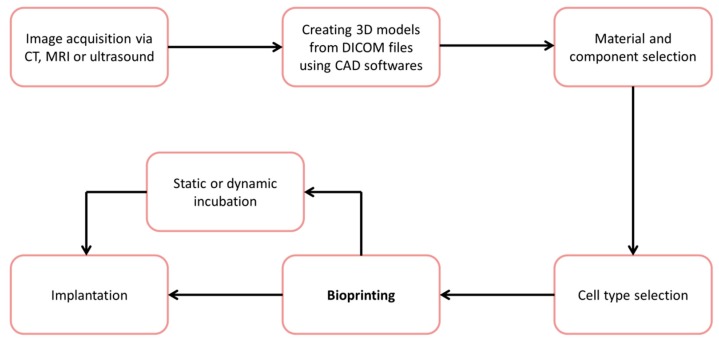
Schematic of Bioprinting Scaffolds for clinical use. Digital 3D images obtained from CT, MRI or ultrasound, are used to design a suitable scaffold with 3D slicing and CAD software; materials from printing are chosen depending upon the application, and can consist of polymers, ceramics, and bioactive components; cells are selected dependent on the application, a bioink can consist of singular or multiple cell types; post-fabrication 3D culture can be used for characterization, assessment and ultimately implantation. 3D printing is both time and cost effective, enabling fast adjustments and implementation of designs [13]. Designs can be made to match exact defect geometries, improving the union between implant and native tissue, thereby enhancing tissue integration [14]. Additive manufactured scaffolds have shown satisfactory accuracy matching the designs [15,16,17]. Different types of tissues and organs have been produced using bioprinting, for instance; blood vessels [18], heart tissue [19], skin [20,21], liver tissue [5], neural tissue [22], cartilage [23] and bone [24].

Bioprinting can be applied in a clinical setting, where it can be used to create regenerative scaffolds to suit patient specific requirements [12]. The process of applying bioprinting to a clinical setting is depicted in Figure 1. To begin with, imaging modalities such as CT, MRI and ultrasound can be used to create a digital 3D model of the tissue defect. Using computer aided design (CAD), the internal and external architecture of the scaffold, such as porosity and pore sizes, can be incorporated into the 3D model of the tissue defect. In consideration of the defect type, location and requirements, a selection of materials, cell types and bioactive molecules, can be used to fabricate a bioink for printing. Cell laden structures are then manufactured using bioprinting technology and are then placed either in cell culture or directly implanted into the patient.

The ultimate aim of bioprinting is to provide an alternative to autologous and allogeneic tissue implants, as well as to replace animal testing for the study of disease and development of treatments. In this review, the main bioprinting techniques are discussed: inkjet-based, extrusion-based and laser-assisted, including their basic mechanisms and current challenges. Table 1, Table 2 and Table 3 provide an overview of recent research for each technique.

An important component of bioprinting is the use of bioinks. Bioinks consist of biomaterials that can be used to encapsulate cells and incorporate biomolecules. Cell laden bioinks are hydrogel-based, as hydrogels have a high water content that is beneficial for cell survivability and shielding the cells from fabrication induced forces. The main properties of a bioink that need to be considered before printing include its viscosity, gelation and crosslinking capabilities. These properties can significantly affect print fidelity (construct stability and print deviation from the computer aided designs) as well as cell viability, proliferation and morphology after printing [25]. To produce a hydrogel that can both support and protect the cells, whilst at the same time provide a structurally secure scaffold is challenging, as these characteristics have different mechanical requirements. Stiff hydrogels have denser networks that might put the cells under pressure during encapsulation, as well as hinder their migration [26]. Ultimately, the hydrogel properties need to be balanced between structural fidelity and cell suspension.

## 2. Inkjet-Based Bioprinting 

**Figure 2:**
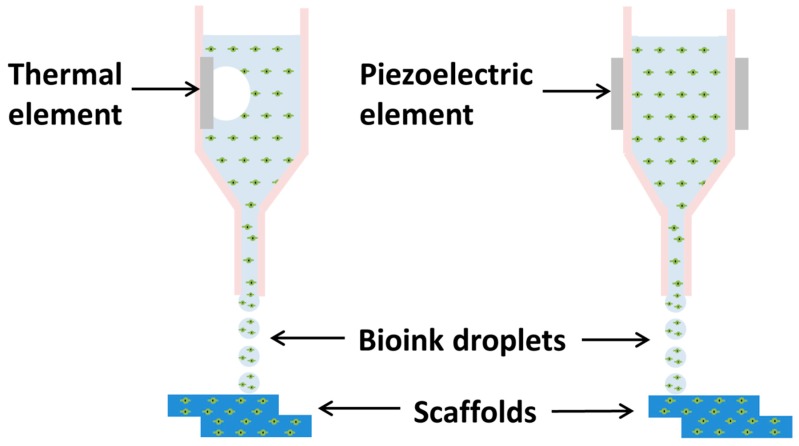
Schematic of Inkjet-based Bioprinting. Thermal inkjet uses heat-induced bubble nucleation that propels the bioink through the micro-nozzle. Piezoelectric actuator produces acoustic waves that propel the bioink through the micro-nozzle.

First attempts to print live cells was performed using a specially adapted commercially available inkjet printers [1]. An initial problem encountered when developing inkjet bioprinting was that the cells died during printing due to instantaneous drying out once on the substrate. The problem was overcome by encapsulating the cells in a highly hydrated polymer, this led to the development of cell-loaded hydrogels [48]. Inkjet bioprinting allows for the precise positioning of cells, with some studies achieving as few as a singular cell per printed droplet [49]. Cells and biomaterials are patterned into a desired pattern using droplets, ejected via thermal or piezoelectric processes, depicted in Figure 2 [1,50]. 

Thermal-based inkjet printing uses a heated element to nucleate a bubble. The bubble causes a build-up pressure within the printhead, which leads to the expulsion of a droplet. The thermal element can reach temperatures between 100 °C to 300 °C. Initially there have been concerns that such high temperatures would damage the cells [51], however research has shown that the high temperatures are localized and are only present for a short time span [11,52].

Piezoelectric-based apparatus uses acoustic waves to eject the bioink. This mechanism limits the use of highly concentrated and viscous bioinks as their viscosity dampens the applied acoustic/pressure waves, hindering the ejection of a droplet [53]. A low viscosity is achieved by using low concentration solutions, a limiting factor for producing 3D structures [50].

Inkjet printing offers a high resolution of up to 50 µm [54]. Most inkjet bioprinters provide a high cell viability, and although there is the potential for induced sheer stresses to damage the cells, most research indicates that this is not the case [55,56]. The advantages of inkjet-based bioprinting include high print speeds, low cost and a wide availability, however problems include low droplet directionality and unreliable cell encapsulation due to the low concentration of the ink [1].

Cui et al. developed a 3D printed bone-like tissue using poly (ethylene glycol) dimethacrylate (PEGDMA), that had a similar compressive modulus to natural bone, and bioceramic nanoparticles [57]. Human mesenchymal stem cells (hMSCs), PEGDMA with hydroxyapatite (HA) and/or bioglass (BG) nanoparticles were bioprinted into bone tissue scaffolds. The bioceramic nanoparticles were used to mimic the native bone tissue microenvironment and stimulated the differentiation of stem cells towards osteogenic linage. There was significant difference between compressive mechanical strengths of pure PEG and PEG-HA scaffolds (~0.35 MPa); however, mechanical strength dropped significantly for PEG-BG scaffolds. Incubation of scaffolds in cell culture for 21 days seemed to increase modulus in all samples except for PEG-BG. The interaction of hMSCs and HA nanoparticles produced highest cell viability of 86% compared to the other scaffolds.

Inkjet bioprinting has demonstrated excellent cell viabilities and the potential for creating a neural network in printed organs. Tse et al. fabricated neural tissue by bioprinting porcine Schwann cells and neuronal NG 108-15 cells using a piezoelectric inkjet printer [32]. Neuronal and glial cell viabilities of 86% and 90% were observed immediately after printing. Proliferation rate of the printed cells was close to those which weren’t printed. The printed cells seemed to have developed neurites that elongated after 7 days. 

Cardiac tissue with a beating cell response was engineered by Aho et al. using feline cardiomyocytes HI.1 cardiac muscle cells and an alginate hydrogel. The tissue was fabricated by printing layers of CaCl_2_ into an alginate hydrogel precursor solution to facilitate crosslinking. The results suggested that cardiac cells attached to the alginate, effectively mimicked the native cardiac ECM. The printed cardiac tissues exhibited contractile properties under mild electrical stimuli [33]. 

Min et al. fabricated full thickness skin models with pigmentation using an inkjet technique [58]. Dermal models was fabricated from fibroblast-laden collagen. After culturing for 1 day in fibroblast medium, keratinocytes were printed on top of the dermal model and put in culture for another day. Melanocytes were then printed onto the model and further cultured in melanocyte medium for 2 h. The entire model was subjected to air-liquid-interface for 4 days. The construct had distinctive epidermal and dermal layers. Keratinocytes reached maturation and melanocytes resulted in freckle-like pigmentation (without chemical or UV stimuli). Sodium carbonate was used for crosslinking.Yanez et al. investigated the wound healing capabilities of bioprinted skin grafts [59]. Skin grafts were fabricated by printing fibrinogen solution onto to a layer of collagen that was laden with human dermal fibroblasts (NHDFs). A subsequent layer of thrombin, laden with human dermal microvascular endothelial cells (HMVECs) was bioprinted onto the fibrinogen. Finally, collagen laden with neonatal human epidermal keratinocytes (NHEKs) was printed onto the fibrin-HMVEC layer. The grafts were incubated for 24 h and transplanted subcutaneously in to the backs of mice. Wounds treated with the bioprinted scaffold had completely healed after 14–16 days, whereas wounds treated without the graft healed in 21 days.

Inkjet bioprinting is of great interest as it exhibits high resolution and cell viability. With this process, accurate position of multiple cell types is possible [49,60]. However, the limitations of vertical printing and restricted viscosities may mean that inkjet bioprinting needs to be combined with other printing techniques for future developments.

## 3. Laser-Based Bioprinting 

**Figure 3:**
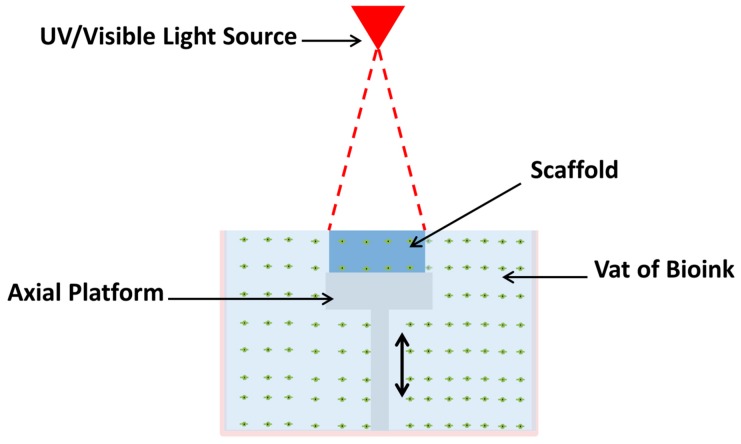
Schematic of Stereolithography Bioprinting. Photopolymerization occurs on the surface of the vat where the light-sensitive bioink is exposed to light energy. Axial platform moves downward the Z-axis during fabrication. This layer-by-layer technique does not depend on the complexity of the design, rather on its height.

Stereolithography (SLA) is an AM technique that uses ultraviolet (UV) or visible light to cure photosensitive polymers in a layer-by-layer fashion, as shown in Figure 3. This nozzle-free technique eliminates the negative effects of shear pressure encountered when using nozzle-based bioprinting. It offers a fast and accurate fabrication, with resolutions ranging between 5–300 µm [61,62]. Polymerization occurs at the top of the bioink vat where the biomaterial is exposed to the light energy. After each layer is polymerized, the platform supporting the structure will be lowered in the vat, enabling a new layer to be photopolymerized on top. 

Photoinitiators are chemical molecules that create reactive agents when exposed to light energy, which react with monomers of a material to then initiate the formation of polymer chains. Photoinitiators are sensitive to different ranges of wavelength; some are triggered by UV and others by visible light. The stiffness and network density of the cured resin depends on the concentration of the photointiator but higher concentrations might exhibit adverse cytotoxic effects. However, different photoinitiators have different cytotoxicity levels. The most commonly used and the least cytotoxic photoinitiators are Irgacure 2959 for UV cross-linkage and eosin Y for visible light [63]. Eosin Y has even shown to be less toxic than Irgacure 2959 [63]. UV light will affect cells and introduce mutations [64]; therefore, visible light-based photocross-linkage has been adopted more frequently in SLA as well as in situ applications [65,66]. Photopolymerization is also employed during or post-fabrication via inkjet- and extrusion-based printing to harden the prints [26,57].

Due to the risk of damaging the cells through the use of UV light or cytotoxic effects of the photoinitiators, several researchers have investigated alternative means to enable photopolymerization of bioinks. Hoffmann et al. developed a class of materials that crosslink without the presence of a photoinitiator using a thiol-ene reaction [67]. The used monomers comprise two classes of monomers containing at least two alkene or thiol groups. These two components react spontaneously under ultraviolet (UV)-irradiation at a wavelength of approximately 266 nm. A 1:1 ratio of thiol and alkene exhibited high cell viability after 3 days, ≈95%. However, doubling the thiol content resulted in a cytotoxic effect, even though this amount of thiol groups provides high amounts of surface functional groups, allowing greater subsequent surface functionalization. 

Zhang et al. used UV laser in the form of Bessel beam [68]. Bessel beam does not diffract and spread out, which will be useful to increase print fidelity and decrease fabrication time. The precursor hydrogel was prepared from GelMA, PEGMA and Irgacure 2959. Human umbilical vein endothelial cells (HUVECs) were encapsulated in the hydrogel. Cell-laden fibers with diameters 25, 43 and 75 µm were fabricated and cell viability was 95% after 3 days. This technique has potential in fabricating tubular constructs and porous scaffolds under a shortened fabrication time; however, is limited to low structural complexity.

Tuan et al. developed a visible light-based stereolithography using Lithium phenyl-2,4,6-trimethylbenzoylphosphinate (LAP) [69], which is a UV-sensitive photoinitiator that can also respond to near-UV blue light [63]. Human adipose-derived stem cells (hADSCs) were suspended in a Poly(ethylene glycol) diacrylate (PEGDA)/LAP solution. Although near-UV blue light, 400–490 nm can be damaging to mammalian cells [70], after fabrication the hADSCs exhibited a high metabolic activity, increasing by 75% and 50% after 5 and 7 days, respectively. 

Other photoinitiators that can absorb visible light are camphorquinone and eosin Y, that crosslink at wavelengths of 400–700 nm and 514 nm, respectively [63]. Wang et al. mixed PEG with eosin Y and methacrylated gelatin (GelMA). Samples without GelMA exhibited decreased cell viability compared to the samples consisting of 5% and 7.5% GelMA, which maintained cell viabilities of ~80% after 5 days [71]. The slightly decreased cell viability could be related to the fact that PEG is non-adhesive, causing the death of anchorage-dependent cells [72,73]. 

Wang et al. fabricated GelMA-based scaffolds via visible light-based SLA [74]. The precursor gel was mixed with eosin Y and NIH-3T3 fibroblasts. The scaffolds were crosslinked by a commercial projector at 522 nm wavelength. After 5 days in culture, most of the cells adhered to bioink. 

Hu et al. studied the cytotoxicity of chitosan-based scaffolds that were mixed with either camphorquinone, fluorescein or riboflavin [75]. Fluorescein and riboflavin are blue light-absorbing initiators. Camphorquinone exhibited relatively low cell viability, ~40%, whilst the other two photoinitiators exhibited cell viabilities >80%. Camphorquinone is more commonly used than the other two photoinitiators; however, biocompatibility results of camphorquinone have been inconsistent in literature [75,76,77].

Stereolithography has much to offer in its application to bioprinting. The absence of shear stress and no limitation on bioink viscosity make it as an appealing choice for incorporating cells within scaffolds. However, the limitations of SLA include the damage caused by UV and near UV light to cell DNA, the limited choice of photosensitive biomaterials as well as the cytotoxicity of added. Some researchers have already begun to look for alternatives, such as using photoinitiator-free materials or visible light-absorbing photoinitiators [67,78].

## 4. Laser-Assisted Bioprinting

**Figure 4:**
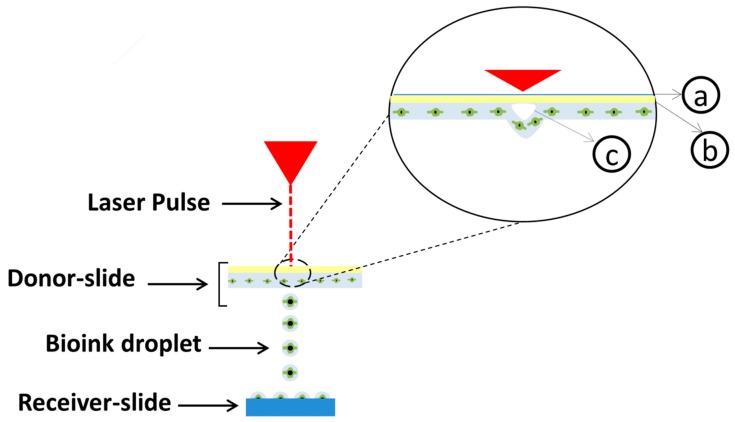
Schematic of Laser-assisted Bioprinting. (**a**) transparent glass, (**b**) thin metal layer, (**c**) vaporization-induced bubble. Bubble nucleation induced by laser energy propels droplets of bioink towards the substrate. This technique has minimal effect on cell viability. A receiver-slide can be a biopaper, polymer sheet or scaffold.

Laser-assisted printing was initially developed to deposit metals onto receiver sheets [79,80]. Odde and Renn later developed the technique to print viable embryonic chick spinal cord cells [81]. Laser-assisted bioprinting (LAB) consists of three parts: a donor-slide (or ribbon), a laser pulse and a receiver-slide. A ribbon is made of a layer of transparent glass, a thin layer of metal, and a layer of bioink. The bioink is transferred from the ribbon onto the receiver slide when the metal layer under the hydrogel is vaporized by a laser pulse, as depicted in Figure 4. This scaffold-free technique has very high cell viabilities (>95 [54]) and a resolution between 10–50 µm [1]. Some studies using LAB have demonstrated an accuracy of a singular cell per droplet [82].

Gruene et al. conducted a study to observe the effects of the LAB laser pulse had on printed mesenchymal stems cells (MSCs). It was found that the laser pulse had a negligible effect. There were no reported changes in gene expression caused by the heat shock of the laser pulse, and cell proliferation rates were as high as the control of non-printed cells after 5 days in cell culture [24]. Alkaline phosphatase (ALP) expression and calcium accumulation were similar to non-printed MSCs after 3 weeks in osteogenic medium. 

Keriquel et al. printed in situ MSCs on to a collagen/nanohydroxyapatite (nHA) disks placed cranial defects [82]. Compared to acellular collagen/nHA disks, the disks with the bioprinted MSC cells exhibited a larger bone volume after 2 months. Michael et al. printed 20 layers of keratinocytes on top of 20 layers of fibroblasts, situated on top of a carrier matrix, Matriderm^®^ that provided stability [21]. Keratinocytes developed into a stratified dense tissue in an in vivo study after 11 days implanted subcutaneously in mice, and demonstrated the potential for LAB in skin tissue regeneration. 

LAB has the ability to position multiple cell types with a high degree of accuracy, with several studies demonstrating singular the capability of positioning a singular cell per droplet [29,81,83]. However, it is an expensive process to perform and suffers from low stability and scalability. It has shown great potential when combined with other biofabrication techniques [29,84].

## 5. Extrusion-Based Bioprinting

**Figure 5:**
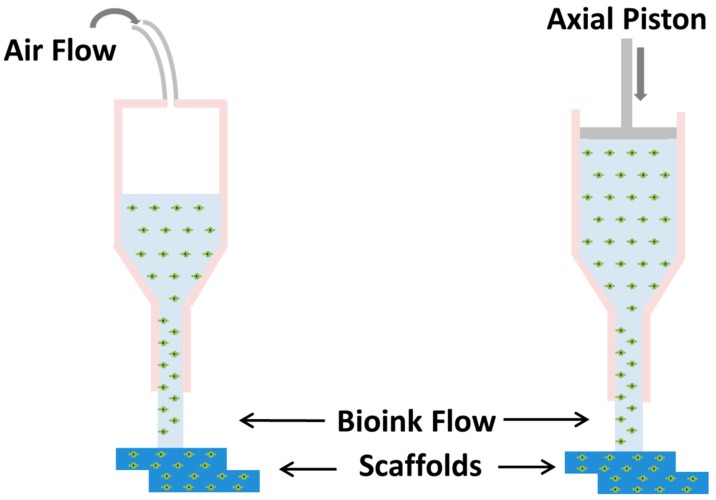
Schematic of Extrusion-based Bioprinting; from left, pneumatic-based and right, mechanical-based. Struts are extruded via pneumatic or mechanical pressure through micro-nozzles. Extrusion-based techniques can produce structures with great mechanical properties and print fidelity.

Extrusion-based printing is a pressure-driven technology. The bioink is extruded through a nozzle, driven either by pneumatic or mechanical pressure, and deposited in a predesigned structure, as depicted in Figure 5 [50]. The main advantage of extrusion bioprinting is the ability to print with very high cell densities [85,86]. Despite its versatility and benefits, it has some disadvantages when compared to other technologies. The resolution is very limited, as a minimum feature size is generally over 100 µm, which is a poorer resolution than that of other bioprinting techniques [87]. This could limit its application for certain soft tissue applications that require small pore sizes for an improved tissue response [11,86,88], however could still be applicable to hard tissues with size larger than 10 mm [35,86]. The pressure used for the extrusion of the material has the potential to alter the cell morphology and function, although several studies have reported [86]. Overall, before printing of the hydrogel can be performed a detailed study with different process parameters including viscosity, nozzle diameter and the accompanied shear stress has to be evaluated [89,90]. This fabrication technique uses highly viscous hydrogel and does not necessarily require any chemical additives for the curing of printed structure [86]. Rheological behavior of the hydrogel ink is very important for extrusion-based bioprinting. Hydrogels are mostly non-Newtonian fluids, meaning that their viscosity changes with shear rate. However, the more viscous the bioink, the higher the induced shear-stress during printing, resulting in higher cell apoptotic activity. An important phenomenon in non-Newtonian fluids is shear thinning, which is a drop of viscosity with an applied shear force. This has a direct impact on the print quality, enabling a plug-like flow to be established, providing greater control over starting and stopping the extrusion process [91]. Although low viscosities result in less dense networks that could allow for better cellular infiltration, too low viscosities will produce a structure that has a poor definition that will ultimately affect print fidelity. 

A study conducted by Chung et al., observed the bioink properties and the printability of alginate-gelatin blends. Using Alg-Gel ink solutions, the printing of scaffolds from three different alginate concentrations (1, 2, and 4% *w*/*v*) were compared. Both printed scaffolds using 2% Alg-Gel and 4% Alg-Gel demonstrated defined structures and maintained their ability to support optimal cell growth. The highly hydrated network structure permits the exchange of gases and nutrients [92]. When choosing a hydrogel to use as the base material, a trade-off must be made between rigidness and softness in order to have a strong supporting structure that allows for nutrient infiltration and the capability to encapsulate cells. High concentrations or crosslink densities are needed to keep a good printing fidelity, yet this limits cell migration. However, low concentrations usually have a poor printability and low mechanical properties. To improve the mechanical properties of the hydrogel, reinforcing fibers like PCL can be used [93]. Photopolymerization is emerging as a promising crosslinking reaction for bioprinting because it enables the rapid formation of hydrogels immediately after printing to maintain print fidelity through the incidence of light energy at appropriate wavelengths [1,94,95,96,97]. The printing resolution can also affected by the diffusion and fusion of the bioinks, which could be solved by reducing the extrusion rate or accelerating the moving speed. With good cell compatibility of the hydrogel material and the high printing quality with appropriate printing process parameters, the hydrogel deposition in the fabrication of tissues or organs can be obtained [98].

An important characteristic for the hydrogel is that it should maintain its mechanical properties after printing. During printing, the hydrogel is subjected to different forces. In nozzle based printing systems, such as with inkjet and extrusion-based techniques, high shear forces can break or disrupt the interlinking bonds of the hydrogel molecular network. This damage to the hydrogel crosslinking can cause a drop in viscosity and a reduction in print fidelity. To overcome this issue, research has been conducted into self-healing hydrogels [99]. A self-healing hydrogel can retain its printed shape due to its non-covalent reversible bonds [100,101]. An improved structure of hydrogels is a structure that has interpenetrating polymer networks (IPNs, which consist of 2 (or more) polymer networks; where one is crosslinked in the immediate presence of another [102]. The networks can be crosslinked simultaneously or sequentially, from heterogeneous or homogeneous materials. An example of IPNs made of heterogeneous materials is double network (DN) IPNs, which is fabricated in a 2-step polymerization process of rigid and soft hydrogels [103]. Biocompatible DNs have been successfully employed in cell encapsulation [104]. 

Cell survivability and function can also be negatively influenced by the extrusion process. In highly concentrated bioinks, shear stresses have the potential to cause cell apoptosis and a drop in the number of living cells [1,86,105]. Shear stress can also affect cell morphology and metabolic activity, as well as the adhesiveness of the cells to the substrate [86]. However, the overall cellular response is dependent upon cell type, as some cells are more resistant than others [86]. 

Extrusion printing can be regarded as a promising technology that allows the fabrication of organized constructs at clinically relevant sizes within a reasonable time frame. However, selection of biomaterial and bioink concentration is important for the survival of the cells during fabrication, as well as the maintenance of cell viability and functionality post-printing. 

Lee et al. used an extrusion bioprinter to regenerate an ear formed of auricular cartilage and fat tissue [106]. The ear shaped scaffold was fabricated using chondrocytes and adipose-derived stromal cells, encapsulated in a hydrogel composed of PCL and poly(ethylene-glycol) (PEG). The bioprinted ear achieved a 95% cell viability [106]. The regeneration of the ear has been considered to be a challenge due to its complex structure and composition, which is difficult to replicate using traditional fabrication techniques. 

Kundu et al. produced cartilage scaffolds by extruding alginate hydrogel onto PCL [107]. Scaffold were printed either with or without human inferior turbinate-tissue derived mesenchymal stromal cells (hTMSCs) within the alginate bioink. Better chondrogenic function was observed when the hTMSCs were encapsulated in alginate gel as well as an increase in extra cellular matrix (ECM) production without an adverse tissue response when implanted into the dorsal subcutaneous spaces of mice [107]. The encapsulation of the cells in alginate hydrogel showed negligible effects on the viability of the chondrocytes which addressed the formation and synthesis of cartilaginous ECM. 

Pati et al. developed a hybrid scaffold combining PCL and decellularized extracellular matrix (dECM) [108]. The dECM bioink was loaded with stem cells derived from adipose, cartilage and heart tissues, and deposited into a PCL framework. It was observed that there was a cell-to-cell interconnectivity within 24 h and a cell and viability of 90% on day 7. This study shows the ability to print complex structures with appropriate material and cells, which can provide an optimized microenvironment that is conductive to the growth of 3D structured tissues. 

Miri et al. demonstrated the possibility to create hierarchical cell laden structures to mimic multicellular tissues [26]. For in vitro studies, hydrogels including poly(ethylene glycol) diacrylate (PEGDA) and methacrylated gelatin (GelMA) loaded with NIH/3T3 fibroblasts and C2C12 skeletal muscle cells were printed into structures resembling musculoskeletal junctions, muscle strips and tumor angiogenesis. The prints retained interfaces and adequate proliferation rates after 3, 5 and 7 days in cell culture. PEGDA-framed chips that had a concentration-gradient of GelMA ranging from 5–15%, were implanted subcutaneously in rats. The result showed formation of the blood vessel network in the bioactive GelMA hydrogels, while the PEGDA served as the frame in the bioprinted multimaterial structure. This novel pneumatic-based process of creating microfluidic devices enabled the printing of different cell suspensions in order to achievemultimaterial devices. 

Extrusion bioprinting is a promising technique to create biomimetic structures to replace tissues and organs. This technique was also efficient in creating microfluidic chips for research applications. Despite its great versatility and feasibility in vertical printing, extrusion-based bioprinting has a relatively limited resolution that does not allow for cell positioning, and requires an advanced hydrogel bioink that maintains cell viability as well as mechanical integrity which has led to the development and use of self-healing hydrogels as well as interpenetrating polymer networks.

## 6. Discussion

3D bioprinting is a relatively new aspect to tissue engineering and has opened the possibility of creating an unprecedented biomimicry, which could ultimately replace the current gold standard of autografts. Biomimicry, in form and function, has great significance in regenerative medicine, drug screening and understanding pathology [109]. In vitro applications have been used to assess pathological and toxicological conditions, as well as implant integration, and offers a methodology with a high-throughput [110]. Biomimetic microfluidic chips have great potential in replacing animal studies for drug and material screening. 

Each bioprinting technique has different requirements for the bioink that can create diverse effects on the encapsulated cells. Inkjet bioprinting provides high resolution and accurate cell positioning. However, it requires the bioink to have a low concentration, which may result in poor structural integrity and inefficient cell encapsulation. This technique has shown great success in creating neural and skin tissues [32,59]. In skin tissue engineering, scaffolds fabricated using inkjet bioprinting have delivered better results when compared to a commercial graft Alpigraf^®^ to repair full thickness wounds in mice [37]. 

Stereolithography offers the possibility of printing cell-laden structures with the shortest fabrication time possible, hence limiting the exposure of the cells to non-physiological conditions. SLA fabrication does not inflict shear stresses upon the cells, unlike in nozzle based techniques, which have the potential to cause cell apoptosis. However, complex designs that include hollow structures (vessels, vasculature or ducts), can become blocked due to remnants of the precursor hydrogel within the printed pores [26]. Another problem with SLA is that surplus bioink is used as fabrication is performed in a vat. That vat is filled with a larger volume of biomaterial, cells and biomolecules than what is needed for the fabrication of the scaffold. 

Extrusion-based printing is the most feasible technique in terms of vertical configuration, although has the lowest reported cell survival among all techniques. The low survivability is due to the shear stress that arises during printing. An important aspect of extrusion printing is its influence on the hydrogel during and after printing. Due to the high shear stresses induced during printing it is possible that the hydrogel could lose its structural integrity. This has led to the development of self-healing hydrogels, which regain their mechanical integrity after the application of shear [111]. Extrusion-based bioprinting has succeeded in creating complex tissue constructs and multi-material microfluidic devices [36,39]. 

A problem encountered by all techniques when using photopolymerization to harden the bioink, is the cytotoxicity of the photoinitiators used and the damage inflicted by UV (10–400 nm) or near-UV blue (400–490 nm) irradiation. However, alternatives to the use of UV light and the use of photoinitiators are under investigation. Visible light-sensitive photoinitiators have reported less cytotoxicity than the most commonly used UV-sensitive photoinitiators [63], as well as an enhanced print fidelity [78].

Post-fabrication, cell-laden scaffolds can be incubated in culture medium to ensure the attachment of cells [112]. Incubation for longer periods (21 days) has resulted in an increase of mechanical strength of the scaffolds due to tissue development [57]. Incubation can be static in cell culture or dynamic using bioreactors. Dynamic culturing can provide continuous infiltrating flow of medium and/or compressive/tensile loading, which is most beneficial for cartilage and bone tissue engineering [113]. 

Current research demonstrates the feasibility and efficiency of using more than one fabrication technique in the manufacturing process. Inkjet printing and LAB have the capability of accurate cell positioning with both of them having achieved the positioning of singular cells per droplet. However, inkjet printing is limited by its ability to produce a 3D architecture, whereas LAB only positions the bioink onto a prefabricated scaffold and is also associated with a high cost. In contrast, extrusion bioprinting has fast fabrication times for large 3D structures, yet has poor cell survivability. Therefore, by combining either inkjet bioprinting or LAB with extrusion printing could provide the ideal combination for producing a scaffold that has both physiologically relevant proportions as well as supports viable cells.

Research has already been implemented combining different printing techniques. In a study by Kim et al., a skin model was fabricated using an extrusion printer to create the main supporting structure and an inkjet printer was used to position dermal fibroblasts and epidermal keratinocytes within the scaffold [114]. The bioprinted scaffold formed dermal and epidermal layers after culturing. Another study combined extrusion printing with stereolithography to create a model for cancer research, where microfluidic devices were fabricated using a digital micro-mirror device and pneumatic extrusion, to understand tumor angiogenesis [26]. In situ applications, where the cell-laden biomaterial is directly deposited into the defect, are also being investigated for accelerated wound healing and bone regeneration, which have demonstrated improved results in comparison to non-cell containing grafts [53,65,66].

Finally, another aspect of bioprinting is its potential to provide prevascularization of the scaffolds. Accurate cell positioning in LAB and inkjet bioprinting techniques could enable a vasculature to be printed into a scaffold. Both techniques have shown promising results in positioning endothelial cells to induce angiogenesis [29,42]. Prevascularization is essential to avoid necrotic failure of the implantation. Other cell positioning research based on inkjet techniques shows great potential in constructing neural networks within large structures [32].

## 7. Conclusions

Additive manufacturing has been heavily applied to tissue engineering over the past decade. Bioprinting enables the production of scaffolds with a homogeneous distribution of cells throughout a scaffold. An organized distribution of different cell types can be positioned within the supporting material, mimicking tissues with multiple cell types or the interface between two tissues. While the choice of material and design impact the viability and proliferation of the printed cells, the different techniques have also shown variable cell activities post-fabrication. Bioprinting is still under development and has many bridges to cross before entering the clinical world, particularly as an in situ direct application. From this brief review, it is concluded that different applications require different fabrication techniques, depending on required resolution, speed, cost, the ability to print vertically etc. Future developments are now concentrating on the combining of techniques to work in a complementary fashion to optimize the process of creating tissue-mimicking structures. 

## Figures and Tables

**Table 1:** Recent in vitro studies. AG—Agarose, SA—Sodium alginate, PLA—Polylactide fibers, GelMA—gelatin methacryloyl, HUVECs—Human umbilical vein endothelial cells, PEGDA—poly(ethylene glycol) diacrylate, ATCC—Mouse neural stem cell lines, BrCa—breast cancer cells, MSCs—marrow mesenchymal stem cells, Nha—nanocrystalline hydroxyapatite.

Biomaterials	Cells	Results	Significance	Reference
**Extrusion-based techniques**				
SA SA/collagen SA/AG	Chondrocytes	Printed SA/collagen scaffold in cell culture showed enhanced cell proliferation, cartilage specific gene expression and cell adhesion.	SA/collagen is a potential bioink base material for cartilage regeneration	Yang et al., 2017 [27]
Alginate PLA fibers	Human chondrocytes	Printed cells showed a high cell viability (80%).	The addition of sub-micron PLA fibers can be used to improve hydrogel mechanical properties	Kosik-Kozioł 2017 [28]
GelMA	HUVECs	Printed cells form lumen- like structure of the endothelium and contracted with an approximate rate of 60 bpm for up to 7–10 days when cultured.	Successfully demonstrated the 3D printing of endothelialized-myocardium-on-a chip.	Zhang 2016 [19]
**Laser-assisted bioprinting**				
Human Osseous Cell Sheets	HUVECs	Printed cell exhibits the formation of tubule-like structures within the biopaper after 21 days of culture.	Demonstration of self-assembled cell sheets for the soft tissue regeneration.	Kawecki 2018 [29]
**Stereolithography**				
PEGDA and GelMA	MCF-7 breast cancer cell, HUVECs, C2C12 skeletal muscle cells, osteoblasts, fibroblasts, mesenchymal cells.	Fabricated structure exhibited high cell viability, proliferation and metabolic activity.	Demonstrated the flexibility of stereolithography for printing different cell types	Miri 2018 [26]
GelMA and graphene nanoplatelets	ATCC	The printed cells had differentiated, produced well-defined architectures and homogenous cell distribution.	Successfully demonstrated the printing neural stem cells	Zhu 2016 [30]
GelMA and nHA	BrCa and MSCs	Printed MSCs secreted macromolecules that promoted BrCa growth.	Successful model for the investigation of post-metastatic breast cancer progression in bone.	Zhou 2016 [31]
**Inkjet-based techniques**				
Cell suspension	Porcine Schwann cells, Neuronal analogue NG108-15 cells	Printed neuronal cells exhibited high cell viabilities as well as earlier and longer neurite growth than unprinted cells.	Can be incorporated into large tissue models to include an established neuronal network before implantation.	Tse 2016 [32]
Alginate	Primary feline adult cardiomyocytes, HL1 cardiac muscle cell line	Cells remained viable in a large scaffold. Scaffold pulsated under electrical stimulation.	Successfully printed myogenic tissue	Xu 2009 [33]

**Table 2:** Recent in vivo studies. Abbreviations: PU—poly(urethane), PCL—poly(caprolactone), hASCs—human adipose-derived stem cells, NSCs—neural stem cells, PEG—poly(ethylene glycol), HUVECs—human umbilical vein endothelial cells, iPSCs—induced pluripotent stem cells, CM—cardiomyocytes, bMSCs—bone marrow-derived mesenchymal stem cells, ROB—rat osteoblasts, TCP—tricalcium phosphates, HMECs—human microvascular endothelial cells.

Biomaterials	Cells	Results	Significance	Reference
**Extrusion-based techniques**				
Hyaluronic acid, Gelatin, Glycerol, Fibrinogen, PU	Human fibroblasts, Human keratinocytes	Subcutaneous implants in rats reduced wound area to <40% after 14 days. Regenerated skin tissue consisted of epidermis and dermis layers	Novel method to fabricate patient-specific tissue construct to reconstruct facial skin wounds	Seol, 2018 [34]
Human decellularized adipose tissue, PCL	hASCs	The scaffolds proved to be adipo-inductive and exhibited adequate tissue infiltration	Demonstration of a clinically viable method of soft tissue regeneration	Pati, 2015 [35]
PU nanoparticles	NSCs	Implanted in adult zebrafish repaired traumatic brain injuries and restored function	3D printing system that does not involve the use of heat, toxic organic solvents, toxic photoinitiators or UV for crosslinking	Hsieh, 2015 [36]
Alginate/gelatin, Alginate/hyaluronic acid, Alginate/Matrigel	INS1E-ß cells, Islets, (human and mouse)	Implanted subcutaneously in mice, exhibited metabolic activity after 7 days	Demonstrates possibility of encapsulating and printing human islets for islet transplantation applications	Yanez, 2015 [37]
Alginate, Fibrinogen, PEG	HUVECs, iPSCs-derived CMs	Subcutaneous implants in NOD-SCID mice developed a vascular network and CMs exhibited maturation after 2 weeks	Demonstrates an advantageous printing design where extruded filament was composed of 2 different inks	Maiullari, 2018 [38]
PCL, Sodium alginate	Rabbit bMSCs, Rabbit chondrogenic bMSCs, Rabbit respiratory endothelial cells	Neocartilage and neovascularization in rabbits after 12 weeks of tracheal implantation	Demonstrates fabrication of an artificial trachea with two cell types via additive manufacturing	Bae, 2018 [39]
PEG, Laponite XLG, Hyaluronic acid	ROBs	Implanted into rat tibias, exhibited new bone formation after 12 weeks	Demonstrates benefit of extruding the scaffold support material and bioink separately, however combined into one printing process	Xinyun Zhai, 2018 [40]
PCL/TCP/Pluronic^®^ F127, PCL/Pluronic^®^ F127	Human amniotic-derived stem cells, Rabbit ear chondrocytes, Rabbit myoblasts	Implanted into rats, scaffolds with different cell types produced: newly formed vascularized bone tissue; vasculature with physiologically relevant mechanical properties; nerve integration	Showed significant improvements compared to acellular scaffolds for myogenic and osteogenic tissues	Kang, 2016 [41]
**Laser-based techniques**				
Collagen	Mouse fibroblasts, Human keratinocytes	Subcutaneous implants in nude mice form multi-layered epidermis and vascularization towards the printed cells, after 11 days	Utilization of a laser-assisted printing process in adding cells to commercially available skin grafts	Michael, 2013 [21]
**Inkjet-based techniques**				
Fibrin	HMECs	Printed cells form confluent tubular structure after 21 days	Promising approach for human microvascular tissue engineering	Cui, 2009 [42]
Collagen, Thrombin, Fibrinogen	Neonatal human dermal fibroblasts and epidermal keratinocytes, Dermal microvascular endothelial cells	Printed scaffolds exhibited 17% better wound contraction after 6 weeks in nude mice	Positioning of microvascular endothelial cells on fibroblast/keratinocyte grafts seemed to be advantageous over commercially available fibroblast/keratinocyte grafts	Marchioli, 2015 [43]

**Table 3:** Recent in situ studies. Abbreviations: IPFP—Human infrapatellar fat pad-derived adipose stem cells, GelMA—gelatin methacryloyl, HAMa—hyaluronic acid–methacrylate hydrogel, PEGDMA—Poly(ethylene glycol) dimethacrylate, AFS—Amniotic fluid-derived stem cells, MSCs—bone marrow-derived mesenchymal stem cells.

Biomaterials	Cells	Results	Significance	Reference
**Extrusion-based techniques**				
HA-GelMA	MSCs	Demonstrated cultured cells directly into the cartilage defect in sheep.	Directly reconstruction of cartilage using extrusion printing.	Di Bella 2017 [44]
**Laser-based techniques**				
nHA	MSCs	Printed cells exhibits the presence of pulsating blood vessels after bone defect achievement.	Scaffold was successfully printed in the mouse calvaria defect model in vivo.	Keriquel 2010 [45]
**Inkjet-based techniques**				
PEGDMA	Human chondrocytes	Printed directly onto the femoral condyles defects showed enhanced tissue integration.	Improved integration by direct in situ printing.	Cui 2012 [46]
Fibrinogen-collagen	AFS and MSCs	Used to repair full thickness wounds in the backs of mice, histological test shows the presence of blood vessel in the subcutaneous adipose tissue.	Potential to quickly close full thickness burns and enable revascularization of the tissue.	Skardal 2012 [47]

## Abbreviations

AFS	Amniotic fluid-derived stem cells
AG	Agarose
ALP	Alkaline phosphatase
AM	Additive manufacturing
ATCC	Mouse neural stem cell lines
BMSCs	Bone marrow stromal cells
BrCa	Breast cancer cells
CAD	Computer aided design
CT	Computer Tomography
dECM	Decellularized extracellular matrix
DN	Double network
DNA	Deoxyribonucleic acid
ECM	Extracellular matrix
GelMA	Gelatin methacryloyl
HA	Hydroxyapatite
hADSCs	Human adipose-derived stem cells
HAMa	Hyaluronic acid–methacrylate
HMECs	Human microvascular endothelial cells
HMVECs	Human dermal microvascular endothelial cells
Hs68	Human dermal fibroblasts
hTMSCs	Human inferior turbinate-tissue derived mesenchymal stromal cells
HUVECs	Human umbilical vein endothelial cells
IPFP	Human infrapatellar fat pad derived adipose stem cells
IPNs	Interpenetrating polymer networks
LAB	Laser-assisted bioprinting
LAP	Lithium phenyl-2,4,6-trimethylbenzoylphosphinate
MRI	Magnetic Resonance Imaging
MSCs	Human bone marrow mesenchymal stem cells
nHA	Nanocrystalline hydroxyapatite
NHDFs	Human dermal fibroblasts
NHEKs	Neonatal human epidermal keratinocytes
PCL	Polycaprolactone
PEG	Poly(ethylene-glycol)
PEGDA	poly(ethylene glycol) diacrylate
PEGDMA	Poly(ethylene glycol) dimethacrylate
PLA	Polylactide fibers
PVA	polyvinyl alcohol
SA	Sodium alginate
SLA	Stereolithography
UV	Ultraviolet
VEGF	Vascular endothelial growth factor
β-TCP	Beta-tricalcium phosphate
